# Supplementary material for: Relationship between locomotive syndrome risk test and height and weight in infancy and toddlerhood: a preliminary study
Source: PeerJ. 2025 Sep 25;13:e20114. doi: 10.7717/peerj.20114 (PMC12476858; doi:10.7717/peerj.20114)
Supplement: Supplemental Information 1 — Pearson’s correlation coefficients. *: p<0.05. [file peerj-13-20114-s001.docx]

**Table S1. Partial correlation between the locomotive syndrome risk test, grip strength, and height/weight at birth, 1 year, and 3 years of age, controlling for sex**

|  | Stand-up test | | Two-step length | | Two-step test value | | GLFS-25 | | Grip strength | |
| --- | --- | --- | --- | --- | --- | --- | --- | --- | --- | --- |
|  | ρ | *P* value | r | *P* value | r | *P* value | ρ | *P* value | r | *P* value |
| Height at birth (cm) | -0.192 | 0.136 | 0.396 | 0.002* | 0.196 | 0.130 | -0.298 | 0.019* | 0.169 | 0.192 |
| Weight at birth (g) | -0.010 | 0.937 | 0.332 | 0.009* | 0.179 | 0.169 | -0.051 | 0.696 | 0.205 | 0.113 |
| Height at age 1 (cm) | -0.342 | 0.007* | 0.244 | 0.058 | 0.032 | 0.806 | -0.101 | 0.433 | 0.111 | 0.394 |
| Weight at age 1 (g) | -0.222 | 0.083 | 0.179 | 0.167 | 0.001 | 0.994 | 0.096 | 0.460 | 0.236 | 0.067 |
| Height at age 3 (cm) | -0.233 | 0.068 | 0.307 | 0.016* | -0.019 | 0.885 | -0.171 | 0.183 | 0.203 | 0.117 |
| Weight at age 3 (g) | -0.213 | 0.097 | 0.189 | 0.145 | -0.051 | 0.697 | -0.078 | 0.545 | 0.304 | 0.017* |

The Stand-up Test and GLFS-25 were assessed using sex-adjusted Spearman’s partial correlation coefficients.

The two-step test value and grip strength were assessed using sex-adjusted Pearson’s partial correlation coefficients.

*: p<0.05.

GLFS-25:25-item geriatric locomotive function scale.

**Table S2. Correlation between height and weight at birth, 1 year, and 3 years of age**

|  | Height at birth | | Weight at birth | | Height at age 1 | | Height at age 1 | | Height at age 3 | | Height at age 3 | |
| --- | --- | --- | --- | --- | --- | --- | --- | --- | --- | --- | --- | --- |
|  | r | *P* value | r | *P* value | r | *P* value | r | *P* value | r | *P* value | r | *P* value |
| Height at birth | – | – | 0.676 | <0.001* | 0.458 | <0.001* | 0.256 | 0.044* | 0.518 | <0.001* | 0.392 | 0.002* |
| Weight at birth |  |  | – | – | 0.378 | 0.002* | 0.317 | 0.012* | 0.404 | 0.001* | 0.355 | 0.005* |
| Height at age 1 |  |  |  |  | – | – | 0.804 | <0.001* | 0.531 | <0.001* | 0.452 | <0.001* |
| Weight at age 1 |  |  |  |  |  |  | – | – | 0.460 | <0.001* | 0.568 | <0.001* |
| Height at age 3 |  |  |  |  |  |  |  |  | – | – | 0.843 | <0.001* |
| Weight at age 3 |  |  |  |  |  |  |  |  |  |  | – | – |

Pearson’s correlation coefficients are presented.

*: p<0.05.
